# Review of Bio-Based Biodegradable Polymers: Smart Solutions for Sustainable Food Packaging

**DOI:** 10.3390/foods13193027

**Published:** 2024-09-24

**Authors:** Maricica Stoica, Cezar Ionuț Bichescu, Carmen-Mihaela Crețu, Maricela Dragomir, Angela Stela Ivan, Geanina Marcela Podaru, Dimitrie Stoica, Mariana Stuparu-Crețu

**Affiliations:** 1 Cross-Border Faculty, “Dunarea de Jos” University of Galati, 111 Domneasca Street, 800201 Galati, Romania; maricica.stoica@ugal.ro (M.S.); angela.ivan@ugal.ro (A.S.I.); geanina.podaru@ugal.ro (G.M.P.); 2 Faculty of Economic Sciences and Business Administration, “Danubius” University, 3 Galați, 800654 Galati, Romania; carmencretu@univ-danubius.ro; 3 Faculty of Physical Education and Sports, “Dunarea de Jos” University of Galati, 63-65 Gării Street, 800003 Galati, Romania; maricela.dragomir@ugal.ro; 4 Faculty of Economics and Business Administration, “Dunarea de Jos” University of Galati, 59-61 Balcescu Street, 800001 Galati, Romania; 5 Faculty of Medicine and Pharmacy, “Dunarea de Jos” University of Galati, 35 Alexandru Ioan Cuza Street, 800010 Galati, Romania; mariana.stuparu@ugal.ro

**Keywords:** environmental impact, biodegradable biopolymers, smart packaging, healthier and safer foods, extended shelf life, consumer acceptance

## Abstract

Conventional passive packaging plays a crucial role in food manufacturing by protecting foods from various external influences. Most packaging materials are polymer-based plastics derived from fossil carbon sources, which are favored for their versatility, aesthetic appeal, and cost-effectiveness. However, the extensive use of these materials poses significant environmental challenges due to their fossil-based origins and persistence in the environment. Global plastic consumption for packaging is expected to nearly triple by 2060, exacerbating the ecological crisis. Moreover, globalization has increased access to a diverse range of foods from around the world, heightening the importance of packaging in providing healthier and safer foods with extended shelf life. In response to these challenges, there is a growing shift to eco-friendly active packaging that not only protects but also preserves the authentic qualities of food, surpassing the roles of conventional passive packaging. This article provides a comprehensive review on the viability, benefits, and challenges of implementing bio-based biodegradable polymers in active food packaging, with the dual goals of environmental sustainability and extending food shelf life.

## 1. Introduction

**Figure 1:**
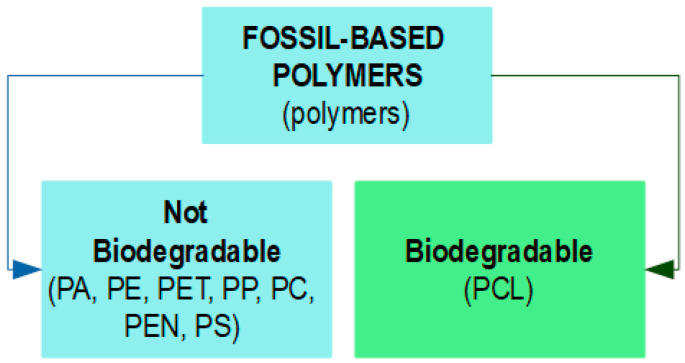
Fossil-based polymers (plastics).

Food packaging is an integral component of food manufacturing, with the materials used in this sector having an essential contribution in protecting food from biological and physicochemical damage, while also ensuring its safety and quality throughout its shelf life [1,2,3,4,5,6,7,8,9,10,11]. The majority of materials used for food packaging are polymer-based (plastics) derived from fossil carbon sources, such as polyethylene (PE), high-density polyethylene (HDPE), low-density polyethylene (LDPE), linear low-density polyethylene (LLDPE), polyethylene terephthalate (PET), polyamide (PA), polypropylene (PP), polycarbonate (PC), polyethylene naphthalate (PEN), polystyrene (PS), and polycaprolactone (PCL), as shown in Figure 1. These materials are favored for their exceptional performance, including aesthetic design, high physicochemical qualities, processability, versatility, as well as their ability to form both flexible structures (bags, films, pouches) and rigid structures (bottles, caps, lids), and for their economic efficiency [1,6,12,13,14,15,16,17,18,19,20].

**Figure 2:**
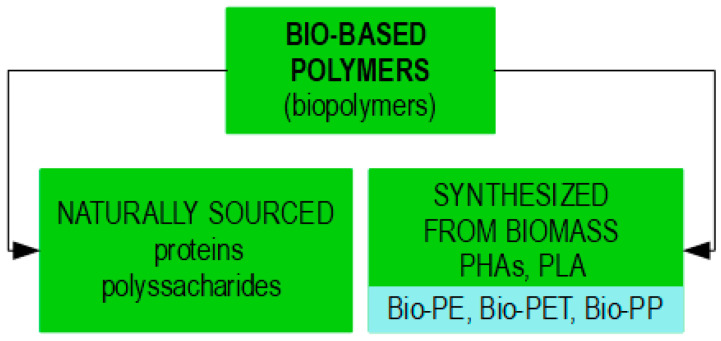
Bio-based polymers (biopolymers).

In 2019, approximately 143 million metric tons of plastics were used globally for packaging. By 2060, this usage is expected to nearly triple, reaching around 380 million metric tons. This increase is particularly significant considering that in developed countries, nearly all food and beverages are sold in packaged form [21,22]. The widespread use of polymer-based packaging, which continues to dominate the food packaging industry, poses a serious threat to global ecological sustainability. The fossil-based origin of plastic materials, along with their longevity, is a key contributor to the ongoing environmental crisis [1,2,6,13,16,17,19,20,23,24,25]. To mitigate the negative impact on the environment and consumer health, the transition to sustainable, green materials for food packaging is essential. This shift aligns with the European Chemicals Agency’s (ECHA) restrictions on the use of plastics [6,8,17,26,27,28]. Due to their low environmental impact, such as renewability, biodegradability, recyclability, and low carbon footprint, bio-based polymers (biopolymers) present a sustainable alternative to fossil-based polymers and offer a viable solution to addressing the plastic crisis [29]. Biopolymers can be classified into two groups: those sourced naturally, such as polysaccharides and proteins, and those synthesized from biomass. The latter group includes both biodegradable biopolymers like polyhydroxyalkanoates (PHAs) and polylactic acid (PLA) and non-biodegradable biopolymers like bio-polyethylene (bio-PE), bio-polyethylene terephthalate (bio-PET), and bio-polypropylene (bio-PP), which are highly resistant to microbial degradation [6,7] (Figure 2).

Although bio-based biodegradable polymers, while environmentally friendly, they may face limitations when used for food packaging applications. They tend to be more susceptible to water than fossil-based polymers and often have poor mechanical and barrier properties. Additionally, their thermal resistance can be inadequate, depending on the specific type of biopolymer [20,30,31]. Furthermore, globalization has made it possible to access a wide variety of foods from any part of the world, making it increasingly important to preserve the authentic sensory qualities of foods (appearance, flavor, smell, texture) [32,33]. In this context, innovative smart packaging (SP) presents a viable solution, by combining elements of intelligent packaging (IP) and active packaging (AP). It is primarily used to assist with the handling, transport, storage, and shipping of foods, as well as to maintain their intrinsic qualities [29,34,35]. IP monitors the quality of packaged foods or their surrounding environment, such as freshness, integrity, storage time, and temperature, and visually communicates this information through digital means. It also aids in tracking the food throughout the manufacturing line and supply chain, all without direct contact with the product itself [29,35,36]. AP goes beyond traditional passive packaging by not only providing a barrier against the external environment but also working to preserve the current state of the food, extend its shelf life, or achieve specific characteristics. This approach opens up new possibilities for enhancing food safety, offering improved protection and quality preservation throughout the food’s shelf life [4,11,24,32,35,37,38,39,40]. In the AP, there is a positive interaction between the package, package headspace, and food products. This is achieved by intentionally incorporating a variety of functional compounds, such as ions, enzymes, bacteriocins, organic acids, and natural extracts into the packaging. These compounds can be placed inside the package, embedded directly into the packaging material, or immobilized on the package’s surface. This approach not only extends the role of conventional packaging, typically designed to be as inert as possible, but also helps to reduce negative environmental impact [2,3,11,14,29,32,41,42,43,44,45,46,47,48,49,50]. The market success of AP ultimately depends on consumer acceptance. Consumers weigh the benefits, such as fresher, safer food with a longer shelf life, against potential risks, including the higher cost of AP-treated food and safety concerns, before making a purchasing decision. In the coming years, investments in AP are expected to rise due to the growth of the food industry, shifts in consumer lifestyles, and digitization of the food supply chain (FSC) [50,51,52,53,54]. This article offers a thorough review of the viability, benefits, and challenges of implementing bio-based biodegradable polymers in active food packaging. It aims to address two key objectives: mitigating the environmental crisis caused by the widespread use of fossil-based packaging and extending the shelf life of food products.

## 2. Bio-Based Biodegradable Polymers

Bio-based biodegradable polymers (biopolymers) are classified into two categories: naturally sourced, such as proteins and polysaccharides, and synthesized from biomass like PHAs and PLA.

### 2.1. Naturally Sourced Polymers

Naturally sourced biopolymers are naturally derived materials, found in high amounts in nature, including animals, plants, algae, and microorganisms [6,55,56,57,58,59]. These biopolymers are an excellent choice for developing food packaging due to their environmental benefits, such as renewability, biodegradability, edibility, affordability, and their potential as sustainable alternatives to fossil-based polymers [15,20,55,57,59,60,61,62]. This sub-section focuses on various biopolymers, such as caseins and whey proteins, collagen and gelatin, chitin and chitosan, keratin, gluten, soy, starch and zein, cellulose, pectin, alginate, pullulan, and kefiran, which are suitable for use as bio-based biodegradable materials in the food and beverage packaging sector. The structures of these biopolymers are given in Table 1.

Animal-sourced biopolymers. Caseins, which make up 80% of milk proteins, possess several beneficial properties including biodegradability, chemical resistance, non-toxicity, the ability to bind small particles and form micelles, strong emulsification capacity, and film-forming ability. These characteristics make caseins ideal materials for developing biodegradable food packaging [23,83,84]. Whey proteins, which constitute 20% of milk proteins, are valuable by-products of cheese and casein production in the dairy industry. They are inexpensive, abundant, and biodegradable biopolymers, which can be used in the development of edible packaging materials. Whey protein-based films and coatings are flexible and transparent, offering superior mechanical quality and water/oxygen permeability compared to polysaccharides and other proteins [23,57,85,86]. Collagen is one of the most successful, cost-effective bio-based materials in the food packaging industry, particularly for meat products. Its mechanical properties, due to its unique protein structure, are stronger than those of polysaccharides [87,88,89,90,91,92,93,94,95,96,97,98,99]. This biopolymer, industrially produced from the extracellular matrix of connective tissues (bones, ligaments, cartilage, tendons, hides, skin, and other biological tissues from various animals), offers numerous benefits such as antioxidant and film-forming capacity, moisture and oxygen barriers, and structural integrity [57,97,98,100]. Gelatin is mostly found in the bones, hides, hooves, and skins of animals and is produced by the hydrolysis and heat denaturation of these tissues [95,98,99]. This biopolymer, which is thermally denatured collagen, is prevalently used in the food packaging sector, especially in sausage casings and edible films/coatings, owing to its eco-friendly nature (renewability; biodegradability); excellent filmogenic qualities; flexibility; excellent aroma, light, water, and oxygen barriers; and low cost [14,57,88,96,99,100,101,102,103,104,105,106]. Chitin and chitosan are obtained from the exoskeleton of crustaceans, as well as from fungi, mushrooms, and other species such as yeast, insects, and fish, with glucosamine being the building block of chitin. Chitin is insoluble in some usual solvents; therefore, it is deacetylated to produce chitosan, which has better solubility than chitin and good film-forming ability. Chitosan-based films are flexible and transparent, offering strong oxygen barrier and mechanical properties. They also possess excellent antioxidant and antimicrobial properties, particularly effective against fungi and algae, with high bacteriostatic activity due to their polycationic nature [107,108,109,110,111,112,113]. Keratin is a fibrous natural protein that occurs in α-helix and β-fold arrangements in animal epithelia tissues such as fish scales, hair, hides, hooves, horns, wool, beaks, toenails, claws, and feathers. It has film-forming capacities and excellent adhesive potential [57,61,95,114,115,116,117,118,119]. As a fiber-reinforcing biopolymer, keratin features an amorphous matrix with crystalline intermediate filaments within its structure. This structural alignment significantly enhances the mechanical properties of keratin-based materials [119]. 

Gluten is an ideal biodegradable biopolymer for film manufacturing, thanks to its unique viscoelastic protein network. This network, which includes hydrogen, hydrophobic, and disulfide bonds, offers excellent gas-barrier properties, high tensile strength, and elongation at break [59,120,121,122]. Soy proteins, naturally, highly renewable, and biodegradable biopolymers offer several advantages due to their composition, which includes a significant level of reactive amino acids such as arginine, cystine, histidine, and lysine, along with a well-balanced amino acid profile. These advantages include flexibility, cost-effectiveness, film-forming ability, high gelling capacity, biodegradability, and oil resistance [15,56,57,123,124,125,126]. Starch, derived from traditional sources like cereal grains (corn, wheat, rice), potatoes, and tapioca; non-traditional crops (beans, peas, lentils); underutilized plants (amaranth, quinoa); and underutilized agricultural by-products, is one of the most versatile natural biopolymers for biothermoplastic food packaging. Its versatility is due to its availability, good film-forming ability, renewability, biodegradability, excellent barrier quality, safe, and economical attributes [58,108,127]. Zein, essentially a by-product of the starch industry, has great potential to use in sustainable food packaging due to its adhesive film-forming potential (the film being heat sealable), good barrier properties (against moisture and oxygen), high thermal resistance, biodegradability, glossy appearance, low permeability, antimicrobial and antioxidant activity, and cost-effectiveness [14,23,55,57,101,103,119,128,129]. Cellulose, the most abundant renewable biopolymer in nature, derived from sources such as plants, recycled paper, agribusiness by-products, wood waste, algae, bacteria, or created enzymatically, can be converted into innovative food packaging materials (films, coatings, hydrogels) due to its nanofibrillar three-dimensional structure, which provides superior chemical, mechanical, and thermal properties [108,130,131,132,133,134]. Pectin is a complex heteropolysaccharide and a green, environmentally friendly alternative due to its high abundance, water-solubility, edible film-forming ability, flexibility, and its effectiveness as a barrier to moisture, oxygen, and aroma. Additionally, it possesses some antioxidant properties [135,136,137]. 

Alginate is an anionic green biopolymer extracted from brown algae and nitrogen-fixing bacteria that has received much attention for its biodegradability, renewability, availability, edibility, and good film-forming capacity. Additionally, its low cost makes it an ideal choice for food packaging films and coatings [138,139,140,141,142].

Pullulan, a linear microbial exopolysaccharide, has remarkable film-forming properties such as oxygen barrier ability, edibility, and biodegradability, making it a sustainable solution to overcome the issues associated with fossil-based polymers [19,124,127,143,144,145,146]. Kefiran, another microbial exopolysaccharide secreted by *Lactobacillus* and various yeast species, is not as widely available as cellulose or chitosan but is an interesting biopolymer for food packaging due to its characteristics, including antioxidant, antimicrobial, and good gelling properties [42,147,148,149,150]. 

### 2.2. Biodegradable Polymers Synthesized from Biomass

These biopolymers are typically synthesized by microorganisms from renewable resources such as polyhydroxyalkanoates (PHAs), or through fermentation or chemical processes from lactic acid or agricultural waste (e.g., corn, beet, rice, and potatoes), in the case of polylactic acid (PLA). They serve as sustainable alternatives to fossil-based materials, offering comparable performance. The chemical structure of these biodegradable biopolymers is presented in Table 2.

PHAs are a group of energy storage materials produced by various bacteria and extremophilic archaea, which store them as water-insoluble inclusions within their cells. The most notable PHAs include poly(3-hydroxybutyrate) [P(HB), the simplest PHA], poly(hydroxybutyrate-co-3-hydroxyhexanoate) [P(HBH)], and poly(3-hydroxybutyrate-co-3-hydroxyvalerate) [P(HBcoHV)]. P(HB) is a biodegradable, optically active biopolymer with properties similar to fossil-based plastics. Incorporating hydroxyvalerate into P(HB) creates P(HBcoHV), a flexible, UV-resistant, and fully biodegradable polyester with lower melting temperature and molecular weight. This makes it suitable for packaging, though it has a reduced water barrier property compared to P(HB) [7,8,17,153,154]. Compared to P(HB) and P(HBcoHV), P(HBH) has shown a wider processing window, better thermal stability, and more promising mechanical performance due to its tailorable composition of both highly crystalline (3HB) and elastomeric (3HH) units [155]. PHAs are highly biodegradable materials that can fully decompose into water (H_2_O), carbon dioxide (CO_2_), methane (CH_4_), or biomass in natural environments and industrial composting facilities. They offer a sustainable alternative for food packaging, with desirable properties such as good chemical and mechanical strength, hydrophobicity, biodegradability, recyclability, compostability, and renewability, making them competitive with petroleum-based polymers [7,8,17].

PLA has gained attention in packaging due to its chemical resistance, excellent transparency, and effective flavor and odor barrier. It offers a relative moisture barrier, high mechanical strength comparable to PE and PET, UV-light resistance, flexibility, and thermoplasticity. PLA is biodegradable, recyclable, and has a lower carbon footprint, but it requires industrial composting for degradation. The importance of PLA packaging lies in its unique qualities for sustainable applications, such as films, food serviceware, containers, cold drink cups, trays, wrapping, bottles, foams, shopping bags, and coatings. Additionally, it is the most cost-effective bio-based material available [7,8,17,156,157,158].

## 3. Limitations of Biodegradable Biopolymers

Unfortunately, the use of bio-based biodegradable polymers is restricted by several disadvantages that limit their ability to compete with conventional fossil-based polymers (Table 3).

Blending with other biopolymers, adding plasticizers, and chemical modifications are viable solutions to improve food packaging design. Moreover, the incorporation of various antimicrobials and antioxidants can enhance the package’s functionality. These strategies support the adoption of biodegradable polymers derived from renewable biomass in the production of engineered active packaging.

## 4. Smart Packaging

**Figure 3:**
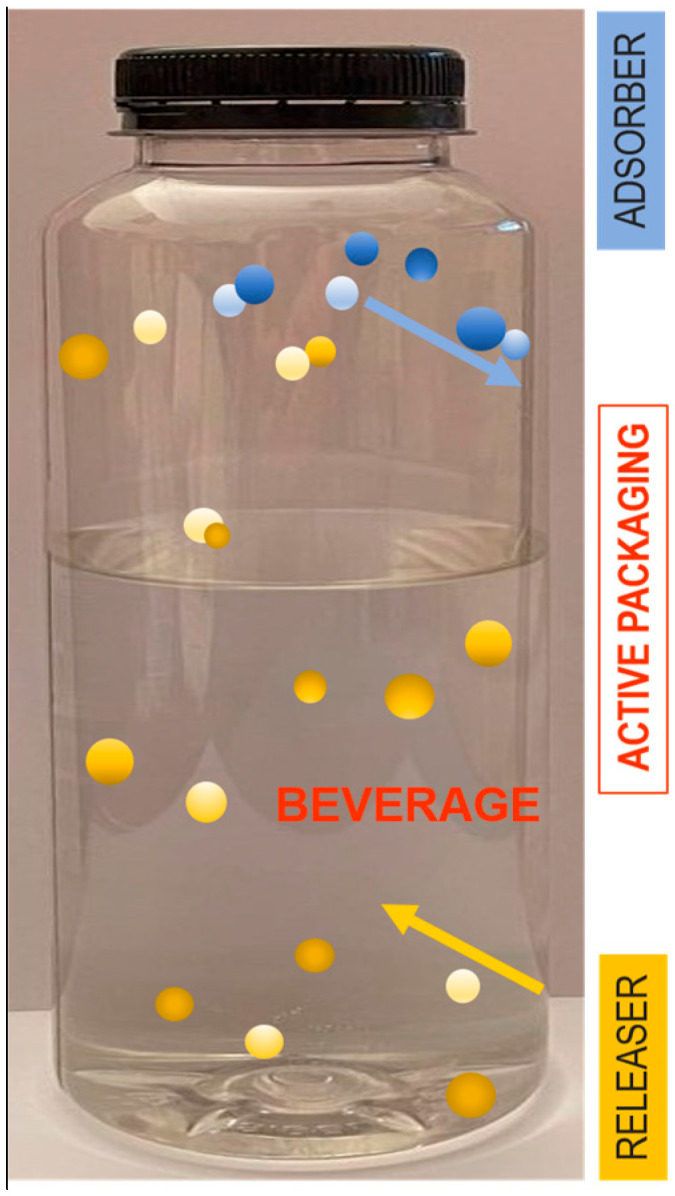
Concept of active packaging in a beverage bottle.

Active packaging (AP) is a new concept specifically designed to either release or absorb compounds into or from the packaged food or beverage (Figure 3), as well as the surrounding packaging environment, with the primary aim of extending the product’s shelf life [29,184,185,186]. 

Figure 3 illustrates the dual function of an AP system, with both adsorber and releaser mechanisms working simultaneously. The packaging itself is active, meaning it interacts with the internal environment of the bottle to enhance the shelf life or quality of the beverage. The blue section labeled Adsorber depicts blue dots being adsorbed from the bottle’s environment. This represents a system that removes unwanted elements such as oxygen, moisture, or other gases from the headspace to prevent beverage degradation. Oxygen absorbers are commonly used to maintain the freshness of beverages. The yellow section labeled Releaser shows yellow dots being released into the beverage. This represents an active releaser system where beneficial substances, such as antioxidants, antimicrobials, or preservatives are released into the beverage to enhance preservation and prolong shelf life. Figure 3 shows the synergy between the adsorber and releaser functions within the packaging. While the adsorber removes detrimental elements, the releaser introduces protective agents, creating a controlled internal environment conducive to extending the beverage’s quality and safety.

### 4.1. AP Releaser/Absorber Systems

AP releaser system contains active compounds with biological properties, such as antimicrobials (CO_2_, nitrogen—N, ozone—O_3_, sulfur dioxide—SO_2_, EOs: eucalyptol, eugenol, cinnamaldehyde, citral, carvacrol, limonene, linalool, vanillin; plant extracts; copper—Cu, silver—Ag, ZnO; carbon dots; bacteriocins, enzymes, organic/mineral acids) or antioxidants (e.g., butylated hydroxytoluene—C_15_H_24_O, butylated hydroxyanisole—C_11_H_16_O_2_, tert-butylhydroquinone—C_10_H_14_O_2_, gallic acid esters, EOs, vegetable oils, vitamin C, vitamin E, carvacol, α-tocopherol, extracts of aromatic plants, carotenoids, nanoliposomes), that are safer and more effective, embedded within the biopolymer matrix [1,4,29,41,43,47,187]. The presence of oxygen (O_2_) in the package headspace initiates unwanted chemical reactions, such as oxidation of pigments, lipids, and proteins by reactive oxygen species (ROS), which negatively impact food quality. These effects include color changes, the development of off-flavors, nutrient losses, and the promotion of microbial growth, particularly aerobic bacteria and O_2_-favored molds, all of which significantly shorten the shelf life of foods [92,188]. One of the most effective methods for preserving the quality of food, especially fresh and highly perishable items, is the use of O_2_ absorbers. These include activated carbon, ferrous iron (FeO), unsaturated hydrocarbons, α-tocopherol, palladium acetate (Pd(CH_₃_COO)_2_), sodium borohydride (NaBH_4_), sodium chloride (NaCl), ascorbic acid, gallic acid, enzymes, microorganisms, and linseed oil encapsulated in silica NPs [44,188,189,190,191,192,193]. O_2_ scavengers are primarily used in various products, including meat products, dairy, bakery, coffee, nuts, snacks, fats, ready-to-eat (RTE) foods, and beverages [3,44,194]. Excessive CO_2_ levels, released during vegetable respiration, can negatively impact food quality and/or the package integrity. To control CO_2_ level, CO_2_ adsorbents can be used, including activated carbon, activated calcium bentonite clay, amino acid salt solutions, anhydrous sodium chloride, biopolymers, calcium hydroxide/oxide, iron powder, sodium carbonate, sodium glycinate, sodium silicate, and zeolite [100,191,195]. These CO_2_ absorbers are commonly used for packaging fresh produce, cheese, meat, poultry, and coffee [3,196]. To control gaseous ethylene (C_2_H_4_) in post-harvest storage and during the marketing of packed fresh fruits and vegetables, various systems can be used, including activated carbon-based systems, potassium permanganate-based systems, palladium-based systems, bentonite-based systems, sepiolite-based systems, and zeolite-based systems [49,100,197,198,199,200,201]. AP is designed to eliminate undesirable off-flavors and odors caused by the oxidation of proteins and lipids or anaerobic glycolysis, which can generate amines, aldehydes, and ketones during the breakdown of foods such as dairy, fish, fruits, and poultry. Various odor absorbers are used for this purpose, including activated carbon, acetylated paper, citric acid, cellulose triacetate, clays, ferrous salts, and sodium bicarbonate [196]. Excessive humidity inside food packaged, particularly fruits and vegetables, often promotes the microorganism’s growth, which reduces the shelf life of the products [41,100]. There are numerous commercial controllers of humidity to manage moisture levels in the food headspace. These include acetylated distarch phosphate, aluminum potassium sulfate dodecahydrate, bentonite, carboxymethylcellulose, modified starch, open-cell expanded PS, PLA silica gel, sodium salt cross-linked, and sodium carboxymethylcellulose, which are typically used for high water activity products like fish, meat, poultry, and produce [41,100,191,202,203,204,205,206,207].

In recent years, meat and meat products have gained increased importance due to their high nutritional value and their role as a significant source of animal protein for humans. However, these products also tend to create favorable conditions for microbial growth, which can lead to spoilage or foodborne illnesses, posing health risks if consumed under unfavorable conditions. Therefore, meat packaging is crucial to ensuring that the products remain in suitable condition along the FSC [208,209,210,211]. The safety and quality of meat are heavily influenced by the packaging materials and technologies used. In this context, smart films and coatings derived from natural sources have garnered significant attention [212]. They not only offer solutions to environmental challenges and provide safer meat products but also intuitively monitor changes in quality and safety, offering consumers dynamic real-time signals about the meat product’s traceability and its quality (freshness, headspace gas composition, pack integrity, etc.). Recent investigations have explored the utilization of smart packaging, particularly its antimicrobial and antioxidant potential, in the meat industry (Table 4).

**Figure 4:**
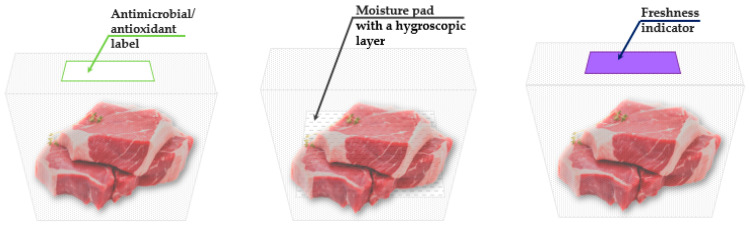
Smart packaging systems for meat products.

Figure 4 visually complements the examples described in Table 4, and it would enhance the understanding of smart packaging systems.

Figure 4 reveals a smart packaging system with an integrated antimicrobial/antioxidant label, designed to protect the quality and extend the shelf life of meat products (on the left side). This label, positioned at the top of the packaging, releases active substances, such as antimicrobial or antioxidant agents, which interact with the contents of the package. By reducing microbial growth and oxidation, the label plays a significant role in maintaining the meat’s freshness and safety over an extended period; an incorporated moisture pad with a hygroscopic layer (in the middle part). This pad is placed at the bottom of the package and is designed to absorb excess moisture released by the meat during storage. The hygroscopic layer actively draws and retains water, preventing the accumulation of liquid in the package. Excess moisture can accelerate microbial growth and degrade the quality of the meat by affecting its texture and promoting spoilage. By controlling moisture levels, the pad helps to maintain the freshness and safety of the meat, prolonging its shelf life; an integrated freshness indicator into the package containing meat products (on the right side). The freshness indicator, located at the top of the packaging, provides real-time visual information about the condition of the meat inside. The indicator’s role is to monitor the quality of the meat by detecting changes in the environment within the packaging, such as variations in gas composition (e.g., O_2_, CO_2_, or volatile organic compounds), which occur as the product degrades. This allows consumers and retailers to assess the freshness of the product at a glance, reducing the risk of consuming spoiled food and enhancing food safety.

### 4.2. Intelligent Packaging

Intelligent packaging (IP), a revolutionary subset of smart packaging, has the potential to revolutionize the food supply chain by monitoring food quality indicators such as freshness, storage time, tightness, and temperature. It involves various components, including indicators and sensors that provide dynamic information (e.g., direct visual changes and detection of specific analytes) and data carriers, which, while not used to collect food quality information, track the movement of food throughout the supply chain [29,35,36,233,234,235].

Indicators visually inform consumers about various properties related to food quality, reflecting the actual conditions to which the food has been exposed and/or its current quality status. They can be thermochromic systems (based on colorimetric or fluorescent dyes), or chemical, enzymatic, microbiological, or mechanical systems, and are typically related to temperature and freshness [233]. Temperature indicators, as indirect indicators of food quality, provide information on whether a critical temperature threshold has been reached during storage and convey the full temperature history of the food throughout the supply chain, rather than directly reflecting changes in the food itself. This alerts the consumers or retailers if food has been exposed to high temperatures. Thermochromic systems indicate temperature through reversible changes, making them more suited for real-time temperature assessment in sensory applications (e.g., cold beverages) rather than for providing information on food stability throughout the supply chain. One of the most practical applications of smart packaging is providing real-time information about food freshness, which helps reduce food waste, improves consumer confidence, and ensures that food is consumed while it remains safe and of high quality. In contrast to temperature indicators, freshness is a direct indicator that offers qualitative or semi-quantitative information on food quality changes caused by physiological or microbiological activity, without compromising the integrity of the food packaging [233,235].

Sensors rapidly and continuously detect specific analytes by converting chemical information into a quantifiable signal (electrical, electrochemical, optical, or gravimetric), which is then processed by electronics and software. These sensors have been proposed for detecting gases produced by food spoilage (e.g., ammonia, hydrogen sulfide) and for monitoring toxic additives or monomers in packaging materials [233,236]. Freshness sensors provide real-time feedback on food freshness by monitoring spoilage markers such as ammonia, pH changes, or microbial activity. A specific type of chemical sensor is the *biosensor*, which incorporates biological materials (e.g., enzymes, antigens, antibodies, nucleic acids) to target specific analytes. Biosensors are particularly used for safety diagnostic, detecting harmful microorganisms in food (e.g., *Escherichia coli*, *Salmonella*), microbial metabolic byproducts (e.g., aflatoxins, biogenic amines—strong indicators of spoilage), allergens, and pesticides [233]. These sensors notify consumers or retailers of potential health risks.

Data carriers (barcodes, QR codes, and radiofrequency identification (RFID) tags) are designed to store and communicate data about a product’s storage history. While typically not used to monitor food quality, they are essential for automation, traceability, theft prevention, and counterfeit protection. These systems can provide real-time updates on expiration status by tracking temperature and other factors throughout the supply chain. Consumers can scan a code to access detailed information about the product’s freshness [233].

Intelligent (responsive) packages are capable of real-time monitoring of food quality by incorporating quality indicators and sensors within the packaging materials to detect changes in key food safety parameters such as pH, temperature, humidity, and the levels (presence, absence, concentration) of target gases like O_2_, CO_2_, nitrogen (N_2_), ethylene (C_2_H_4_), and hydrogen sulfide (H_2_S) [233,234] (Table 5). 

### 4.3. Consumer Views on Active Packaging

Active packaging (AP) market is expected to grow, driven by the expanding food industry, shifting consumer lifestyles, and the digitization of FSC. The expanding food industry presents significant opportunities for the global AP market, with ongoing product innovation expected to drive growth. AP market, valued at $27.19 billion in 2023, is expected to reach $64.64 billion by 2032. However, rising raw material prices and production costs may pose challenges to market expansion [52,247]. The market success of AP ultimately depends on consumers. While they do not directly purchase packaging, their food choices are greatly influenced by both the product and its packaging [51,248]. The functionality and communication function of packaging, which is essential for both consumers and manufacturers, is one of the key factors that influence consumer purchasing decisions [32,54,100]. Food consumers’ purchasing behavior is complex, as they weigh benefits and risks before deciding. Barriers such as high costs, technology neophobia, and lack of information can hinder AP acceptance [50,53,54]. A significant barrier to AP adoption is its high cost compared to conventional packaging, which can significantly increase the final cost of foods, sometimes doubling it, whereas conventional packaging rarely exceeds 10% of the final cost [100]. Consumer preferences for AP technologies also vary, with scavenger technology being more favored over releaser technology. Safety is another concern, as AP materials, unlike conventional packaging, interact with food through migration, posing potential health risks. Additionally, accidental breakage of the sachet/bag and ingestion of AP components are potential hazards [3,49,54]. Europeans have been resistant to innovations in food packaging, having no affinity with AP, while people in Asia, Africa, and South America tend to favor traditional packaging methods, such as using vegetable leaves, due to their natural benefits. A lack of consumer knowledge is a significant barrier to AP adoption. Without understanding its benefits and how it works, consumers may be skeptical. Providing clear information on packaging could help boost acceptance [100].

## 5. Conclusions

Packaging has a crucial role in protecting foods from damage and external influences. Fossil-based polymers are widely used in the food industry as packaging materials due to their advantageous properties, such as clarity, aesthetic appeal, good barrier and mechanical characteristics, versatility, combinability, processability, and cost-effectiveness. However, the excessive use of polymer-based packages, still dominant in the food packaging sector, poses a serious threat to ecological sustainability around the globe. The nonrenewable nature, longevity of these materials, and the expected nearly tripling of global plastic consumption by 2060 are significant contributors to the exacerbation of environmental crises. In recent years, the packaging design has shifted its focus from conventional passive packaging to eco-friendly active packaging, which meets consumers’ demands for healthier and safer foods, rather than merely providing protection from the external environment as conventional passive packaging does. In this context, natural biodegradable biopolymers, due to their unique physicochemical properties, present a promising alternative to fossil-based polymers, offering a sustainable solution for eco-friendly active packaging. Unfortunately, these biopolymers have some drawbacks, such as extreme vulnerability to water and inferior mechanical, barrier, and thermal properties. Blending them with other materials is one approach to designing more effective food packaging. Additionally, the incorporation of antimicrobials and antioxidants can enhance the functionality of these films. These enhancements contribute to the successful implementation of biodegradable biopolymers in engineered food packaging and help mitigate the environmental crises associated with the widespread use of fossil-based packaging.

## Figures and Tables

**Table 1:** Structure of naturally sourced polymers (biopolymers).

Name	Structure		Refs.
Caseins	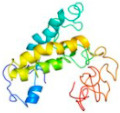	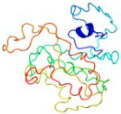	[63]
	α-casein	β-casein	
Whey proteins	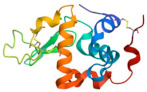	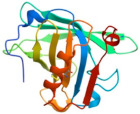	[64,65]
	α-lactalbumin	β-lactoglobulin	
Collagen and gelatin	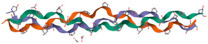	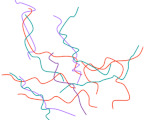	[66,67]
	collagen	denaturated gelatin	
Chitin and chitosan	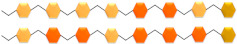		[68]
Keratin	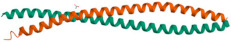		[69,70]
Gluten	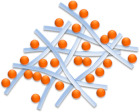		[71,72]
Soy proteins	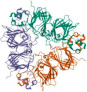		[73,74]
Starch	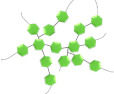		[75]
Zein	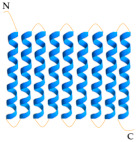		[76]
Cellulose	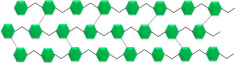		[77]
Pectin	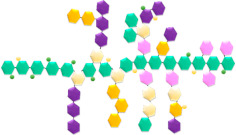		[78]
Alginate	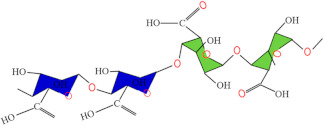		[79,80]
Pullulan	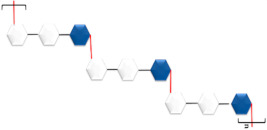		[81]
Kefiran	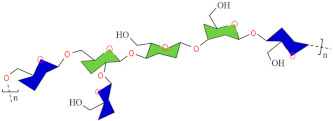		[82]

**Table 2:** Chemical structure of biodegradable biopolymers synthesized from biomass.

Name	Structure		Refs.
PHAs	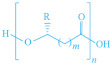	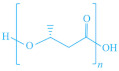	[151]
	PHA	P(HB)	
	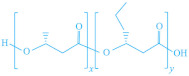	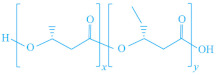	
	P(HBH)	P(HBcoHV)	
PLA	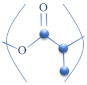		[152]

**Table 3:** Biodegradable biopolymers—limitations and solutions.

Biodegradable Biopolymers	Limitations	Solutions
Caseins	Extremely sensitive to moisture, which severely affects their mechanical characteristics [23]	Cross-linking treatment with divalent cations, which leads to a more stable structure [83]
Whey proteins	Poor tensile strength and moisture resistance due to high amounts of hydrophilic amino acids in the chain of milk proteins [57,83]	Incorporation into the matrix: glycerol, unmodified Na^+^-montmorillonite, other biopolymers (zein, sodium caseinate, nanocelluloses), EOs, or various methods of cross-linking [23,83,159,160,161]
Collagen	Poor wet mechanical properties due to its poor moisture resistance and low thermal stability [57,89,96]	Use of plasticizers (glycerol), suitable cross-linking treatments, blending with other biopolymers (chitosan), and the addition of active compounds [96,162,163,164,165,166]
Gelatin	Poor mechanical properties and strong sensitivity to moisture, tending to swell and dissolve when in contact with food with great humidity levels [57,88,102,105,106]	Cross-linking or combining with other biopolymers (carboxymethyl cellulose, chitosan, soy protein isolate, starch) [88,167,168]
Chitin	Insoluble in some common solvents and has poor biodegradability due to its high crystallinity and high content of acetamido groups [107]	Deacetylation under alkali conditions to produce chitosan [110,111,112]
Chitosan	Poor mechanical properties, barrier performance, and water resistance characteristics due to the presence of many hydrophilic groups in its structure [110,169]	Blending with other biopolymers [110,169]
Keratin	Hydrophobic compound, not suitable as a packaging material in its pure form [57,106]	Proteolytic cleavage by enzymes [61,115,116,119]
Gluten	Pronounced solubility in water, high water attraction, rigid structure, and opacity [23,43,122,170]. Additionally, human intolerance to gluten is one of the biggest disadvantages of gluten-based edible films [112,120]	Incorporation into the matrix: pectin, carboxymethyl cellulose, or other proteins [170]
Soy proteins	High water-solubility, poor mechanical properties, low tensile strength, low thermal stability, reduced transparency, and low heat resistance [15,23,57,126,128,171]	Combining with other biopolymers (chitosan, gelatin, nanocellulose, etc.), plasticizers (glycerin), lipids, and plant extracts [1,126,172,173,174]
Starch	Inferior water resistance and mechanical qualities [108,127,147]	Adding plasticizer (glycerol, sorbitol, sugars), blending with bioactive compounds and other biopolymers (gelatin, pectin, pullulan), and reinforcing with bacterial nanocellulose, metal-oxides, and nanoclay [28,59,108,140,175,176]
Zein	Poor mechanical and thermal qualities, and low water resistance, making it unsuitable for use as food packaging films in its pure form [43,57,128,129]	Blending with other biopolymers (chitosan, pullulan, gelatin, carrageenan, cellulose, alginate, PHAs, soy protein, whey protein), adding plasticizers (glycerol, polyethylene glycol, sorbitol), or incorporating NPs [43,120,128]
Cellulose	Sensitive to water, with reduced mechanical strength and limited barrier characteristics [131]	Incorporation of resins, wax, and reinforcing agents (clay, metal-based NPs, nanocellulose); coating with surfactants; blending with other biopolymers (gelatin, zein); and chemical modifications (acylation, esterification, grafting, and silylation) [55,131,177]
Pectin	Brittle and more hydrophilic, with poor mechanical properties [135,137]	Adding plasticizers (glycerol, sorbitol, sucrose, polyethylene glycol, mannitol), embedding pectin with other polysaccharides (agar, carrageenan, pullulan, chitosan), proteins (gelatin), or synthetic biopolymers (PLA) [135,137]
Alginate	Strong hydrophilicity, limited antimicrobial and antioxidant characteristics and UV-light barrier, and instability under heat treatment [139,151]	Blending with other biopolymers (chitin, chitosan, carboxymethyl cellulose, fish scale gelatin, pectin), embedding NPs embedding (nanosilver, montmorillonite, TiO_2_), incorporating plant extracts, yeasts, or bioactive compounds (carotenoids, vitamin C, phenolic substances), and chemical modifications (amidation, esterification, sulfation, oxidation, and reductive amination) [139,142,178,179]
Pullulan	High hydrophilicity, poor mechanical properties, and limited antioxidant and antibacterial potential [19,124,127,147]	Blending with other biopolymers (alginate, starch, chitosan, zein), embedding of organic/inorganic NPs (e.g., ZnO), or chemical modifications (esterification, oxidation, etherification, sulfation, and amination) [19,127,145,146,180,181,182]
Kefiran	Poor mechanical characteristics [148]	Combining with other biopolymers (carboxymethyl cellulose, starch, chitosan, whey proteins), adding plasticizers (glucose, sucrose, glycerol, lipids), or incorporating reinforcing agents (montmorillonite, nanocellulose, CuO, TiO_2_, ZnO) [148]
PHAs	Inferior thermal and mechanical stability, poor moisture and gas barrier properties, higher aroma permeability, and high cost [8,155,156]	Addition of NPs (nanocellulose, nanoclays, nanosilver, and metal-oxides in nanoforms) [8]
PLA	Brittleness, low gas and vapor barrier properties, low flexibility, thermal instability, and a slow biodegradation rate that can take up to 3–5 years [8,156]	Blending with other biopolymers: poly(butylene succinate), poly(butylene succinate-co-butylene adipate), poly(butylene adipate-co-butylene terephthalate), and PHAs [183]

TiO_2_—titanium dioxide (nanoform), ZnO—zinc oxide (nanoform), CuO—copper oxide (nanoform), NPs—nanoparticles, EOs—essential oils.

**Table 4:** Illustrative examples of smart materials with antioxidant and antibacterial properties used in meat and poultry packaging.

Engineered Films or Coatings	Applications	Refs.
PLA/chitosan	Ready-to-eat deli turkey meat	[213]
Whey protein isolate/oregano/clove essential oil	Chicken breast fillets	[214]
Zein/lysozyme/EDTA	Ground beef patties	[215]
Chitosan/ZnO	Raw meat	[216]
Tapioca starch/grape pomace	Ready-to-eat chicken deli meat	[217]
Okra mucilage/ZnO	Chicken breast meat	[218]
Starch/gallic acid/chitosan/carvacrol	Ham product	[219]
Cellulose/ZnO/gelatin	Chicken fillets	[220]
Cellulose/wheyprotein/TiO_2_/rosemary essential oil	Lamb meat	[221]
Gelatin/chitosan/ZnO	Meat beef	[222]
Pullulan/chitosan/ZnO	Pork belly	[223]
Zein/tea tree essential oil/blueberry anthocyanin	Pork products	[224]
Gelatin/alizarin/oregano essential oil	Beef freshness	[225]
Collagen/delphinidin	Casings in the meat industry	[226]
Collagen/chitosan/gallic acid	Pork	[91]
Gelatin/alginate	Raw minced beef meat	[227]
Gelatin/pullulan/cinnamon essential oil	Meat	[228]
Chitosan/cinnamaldehyde	Handmade meat patties	[229]
Gelatin/chilli seed oil	Fresh chicken breast cubes	[230]
Chitosan/ZnO	Refrigerated poultry meat	[231]
Cellulose/carbon dots	Minced pork	[232]

EDTA—disodium ethylenediaminetetraacetic acid.

**Table 5:** Food safety parameters. Intelligent detectors/controllers [41,99,187,196,200,233,234,235,237,238,239,240,241,242,243,244,245,246].

Parameters	Brief Introduction	Detectors/Controllers
pH	During food storage, both aerobic and anaerobic microorganisms can proliferate, producing organic acids (lactic acid, acetic acid), which lower the pH of food. Additionally, CO_2_, a byproduct of microbial growth, can dissolve in food products, forming carbonic acid that further reduces the pH.	Synthetic pH-sensitive dyes (e.g., bromocresol green, methyl red) Natural pH-sensitive dyes (e.g., anthocyanins, betalains, carotenoids, chlorophyll, curcumin) embedded into biodegradable films (e.g., cellulose, chitin, chitosan, gelatin, and starch), which offer additional antibacterial and antioxidant benefits.
O_2_	O_2_ in the package headspace can initiate undesirable chemical reactions in numerous foods, especially fresh and highly perishable foods. This leads to quality deterioration through color changes, off-flavors, microbial growth, and nutrient loss.	Smart technologies include O_2_ scavengers (to maintain low O_2_ levels inside the package), O_2_ luminescence-based indicators, and colorimetric redox sensors that display color changes to signal when O_2_ levels exceed safe limits. In high-moisture environments typical of food packaging, redox dyes may leach from the water-insoluble polymer matrix, raising health concerns. Alginate’s cation-binding properties have been used to mitigate dye leaching.
CO_2_	High levels of CO_2_, produced during the respiration of fresh produce or through modified atmosphere packaging, can adversely affect food quality and packaging integrity.	Luminescent dyes (e.g., 8-hydroxypyrene-1,3,5-trisulfonic acid in polymeric films) offer high sensitivity but they do not come in contact with the foods, being unsuitable for consumer use. Safer alternatives include colorimetric indicators that detect pH changes caused by CO_2_ hydrolysis, although they offer lower sensitivity (e.g., L-lysine, poly L-lysine, anthocyanins).
N_2_	Animal-derived foods are highly susceptible to the growth of pathogenic microorganisms, which can lead to the formation of biogenic amines, posing a potential safety risk to human health. The total volatile basic nitrogen level (TVB-N) is commonly used as an indicator of spoilage and the production of harmful biogenic amines.	A rapid wireless sensor based on a hydrogel-coated pH-electrode, sensitive to volatile amine levels, provides a highly sensitive response to spoilage. However, integration into packaging systems remains challenging. The sensor changes color or sends an alert when amines are detected.
C_2_H_4_	Ethylene, a volatile plant hormone, released by fruits and vegetables during ripening, can accelerate the ripening process in both climacteric and non-climacteric produce. This leads to reduced shelf life and affects post-harvest storage and marketing.	Smart technologies incorporate C_2_H_4_ scavengers (to maintain low C_2_H_4_ levels inside the package) and C_2_H_4_ nanotechnology sensors, which can alert consumers to the ripening stage of fruits and vegetables, facilitating timely consumption.
H_2_S	Hydrogen sulfide (H_2_S), a volatile produced during enzymatic hydrolysis of sulfur-containing amino acids (e.g., cysteine, homocysteine, and methionine), is a reliable marker for assessing meat freshness.	Colorimetric sensors based on gellan gum-capped silver nanoparticles Nano-bionic sensor using ruthenium nanoparticles Chemical sensor based on supramolecular nanorods synthesized via copper ions
Humidity	Excessive humidity in packaged foods can promote bacterial and fungal growth, degrading nutritional and sensory qualities. Conversely, low humidity levels may cause food dehydration and reduce shelf life.	Wireless humidity sensors, consisting of a planar inductor and capacitor on a paper substrate, can be easily integrated into packaging. However, substrate moisture absorption can alter capacitance. Other options include RFID-coupled humidity sensors and photonic crystal-based humidity sensors.
Temperature	Temperature fluctuations can significantly affect food stability, particularly in refrigerated and frozen products. Temperature abuse can degrade food texture and promote the growth of psychrotrophic bacteria.	Devices in direct contact with food, such as thermochromic inks or sensors, alert consumers and supply chain stakeholders when products are exposed to unfavorable temperatures, helping prevent the sale or consumption of spoiled items. Smart packaging that combines temperature and humidity data with the expected shelf life of the product can more accurately predict expiration dates.

## Data Availability

No new data were created or analyzed in this study. Data sharing is not applicable to this article.
